# Efficiency and safety of sofosbuvir in Bangladeshi children with chronic hepatitis C virus infection

**DOI:** 10.1016/j.iliver.2023.06.002

**Published:** 2023-07-28

**Authors:** Salahuddin Mahmud, Jahida Gulshan, Md. Belayet Hossain, Madhabi Baidya, Rafia Rashid, Farhana Tasneem, Ahmed Rashidul Hasan, Tanzila Farhana, Mohammed Reaz Mobarak, Md. Jahangir Alam, Syed Shafi Ahmed

**Affiliations:** a Department of Pediatric Gastroenterology, Hepatology & Nutrition, Bangladesh Shishu Hospital & Institute, Dhaka, Bangladesh; b Institute of Statistical Research and Training (ISRT), University of Dhaka, Dhaka, Bangladesh; c Department of Pediatric Hematology & Oncology, Bangladesh Shishu Hospital & Institute, Dhaka, Bangladesh; d Department of Pediatric Gastroenterology, Dr. M R Khan Shishu Hospital & Institute of Child Health, Dhaka, Bangladesh; e Department of Pediatrics, BIHS General Hospital, Diabetic Association of Bangladesh, Dhaka, Bangladesh; f Department of Epidemiology & Research, Bangladesh Shishu Hospital & Institute, Dhaka, Bangladesh; g Bangladesh Shishu Hospital & Institute, Dhaka, Bangladesh

**Keywords:** Hepatitis C virus, Direct-acting antivirals, Sofosbuvir, Ribavirin

## Abstract

**Background and aims:**

Currently, treatment with oral direct-acting antivirals is recommended for all hepatitis C virus (HCV)-infected pediatric patients. The aim of this study was to evaluate the efficacy and safety of sofosbuvir and ribavirin combination therapy for children and adolescents in Bangladesh who are living with chronic HCV infection.

**Methods:**

An experimental study was performed from January 2021 to December 2022. HCV polymerase chain reaction (PCR)-positive thalassemic children, who were 6–18 years of age, were enrolled by consecutive nonprobability sampling. Clinical features were recorded, and investigations were performed. All patients were initially treated with sofosbuvir (200 mg for 6- to 11-year-olds and 400 mg for 12- to 18-year-olds) and ribavirin (10–15 mg/kg/day) and were assessed clinically on a four-weekly basis, along with liver-function testing. The total duration of therapy was 24 weeks. HCV PCR was done at the end of treatment and 12 weeks after the completion of treatment to see the sustained virological response.

**Results:**

There were 26 cases in total, with a mean age of 9.26 ± 2.82 years; 14 were males (53.8%), and 12 females (46.2%). Twenty-five (96.15%) patients achieved a sustained virological response, and the end-of-treatment PCR was negative. One patient (3.85%) was a nonresponder even after 24 weeks of treatment. The medication was well received, with only four patients (15.3%) reporting headaches that were reported untreated.

**Conclusion:**

The combination of sofosbuvir and ribavirin is effective in treating chronic HCV infection and is not accompanied by any major negative side effects.

## 1 Introduction

Hepatitis C virus (HCV) is a hepatotropic RNA virus in the Flaviviridae family [1]. It is prevalent in the world's population at endemic proportions, with an estimated overall disease burden of 184 million [2,3]. Infection with chronic HCV affects between 2.1 and 5 million children under the age of 15 worldwide [4,5]. Chronic HCV infection in children is frequently asymptomatic or mildly symptomatic. However, end-stage liver disease necessitating a liver transplant can progress to substantial fibrosis, cirrhosis, hepatocellular carcinoma, and other conditions. It is crucial to detect chronic HCV infection in children and adolescents as early as possible so that treatment can be initiated to reduce the risk of long-term complications [6,7].

The estimates of HCV seroprevalence in children and adolescents vary globally. In Europe and the USA, the prevalence is generally low, with estimates of up to 0.4%, but in Egypt it is higher, at up to 6% [[8], [9], [10]]. HCV is a serious issue in Southeast Asia. In Bangladesh, the prevalence of HCV infection is 0.88%, while the estimated prevalence in Pakistani children is 0.58%. As a result, it is quickly becoming one of Bangladesh's most pressing health concerns [[11], [12], [13]]. Every year, an increasing number of people lose their lives to HCV-related illnesses. Hepatitis C is responsible for approximately 399,000 deaths annually, mainly from cirrhosis or hepatocellular carcinoma. It was estimated that there were 333,000 fatalities in 1990, 499,00 in 2010, and 704,000 in 2013. These numbers come from the Global Burden of Disease study. The high hepatitis C prevalence is reflected in the rising death toll [3,[14], [15], [16], [17]].

Previously, there were fewer therapeutic choices for children with chronic HCV infections than for adults. Standard therapy involves interferon or peginterferon and ribavirin, for 24 or 48 weeks, a regimen that requires subcutaneous injections and is associated with significant side effects, including growth impairment [9,18,19]. Because of safety concerns, poor tolerability, the parenteral route of administration, bone marrow suppression, growth impairment, and poor efficacy against HCV genotypes with peginterferon and ribavirin, there is no consensus on whether or when to use these medications to treat children with chronic HCV infection [2,9,18]. By contrast, the standard therapy for chronic HCV in adults includes several all-oral regimens with direct-acting antivirals (DAA) specifically targeting HCVs that are highly effective and have a favorable safety profile [9,20,21]. It has been suggested that for most children with chronic HCV infection, treatment should be deferred until interferon-free regimens are available [9,22].

In 2017, sofosbuvir with ribavirin and ledipasvir/sofosbuvir were approved to treat HCV in adolescents aged 12–18 years, and, in some regions, younger children weighing at least 35 kg were eligible for treatment [7,[23], [24], [25], [26]]. In 2018, the American Association for the Study of Liver Disease and the Infectious Disease Society of America recommended sofosbuvir with ledipasvir for genotypes 1, 4, 5, and 6 and sofosbuvir with ribavirin for genotypes 2 and 3 in the pediatric population aged 3–18 years [27]. Sofosbuvir is a promising therapeutic agent that inhibits viral replication by binding to the NS5B RNA-dependent RNA polymerase, has a high-sustained virological response rate against different HCV genotypes, and has a 90% cure rate [2].

Comprehensive data regarding the efficacy and safety of sofosbuvir in treating chronic HCV infection is lacking, especially in the pediatric population of Bangladesh. In this study, we evaluated the efficacy and safety of sofosbuvir with ribavirin in the treatment of chronic HCV infection among multitransfused thalassemic Bangladeshi children.

## 2 Materials and methods

### 2.1 Study design

A prospective study was performed in the Department of Pediatric Gastroenterology, Hepatology, and Nutrition at Bangladesh Shishu Hospital & Institute, Dhaka, Bangladesh, from January 2021 to December 2022. A total of 26 admitted cases of chronic HCV infection in thalassemic children between 6 and 18 years of age (referred from the pediatric hemato-oncology department of the same hospital) were enrolled after obtaining informed written consent from their parents. The study was approved by the Ethical Review Committee of Bangladesh Shishu Hospital & Institute, Dhaka, Bangladesh.

### 2.2 Inclusion and exclusion

**Fig. 1:**
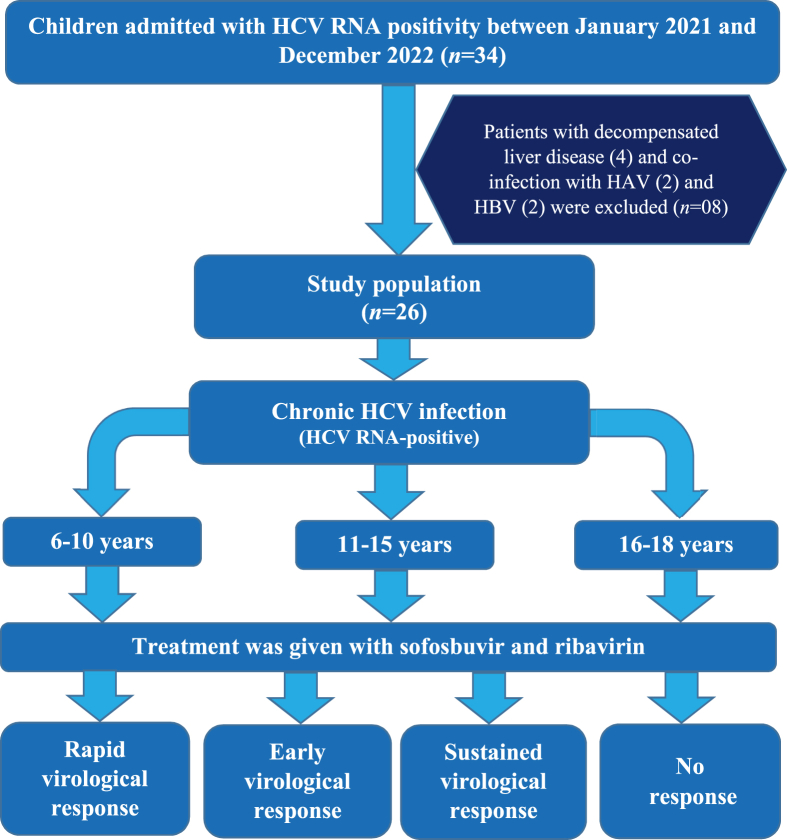
Study flow chart.

HCV PCR-positive children, aged between 6 and 18 years, who had not received any HCV-related treatment were enrolled in the study using a nonprobability sample. Patients younger than 6 years and older than 18 years with decompensated liver disease or acute or chronic liver failure and coinfection with hepatitis A virus, hepatitis B virus, or human immunodeficiency virus were excluded from the study. Information was gathered using a structured questionnaire. Clinical observations including clubbing, leukonychia, palmar erythema, thenar–hypothenar wasting, spider nevi, testicular atrophy, hepatosplenomegaly, and ascites were recorded (Fig. 1).

### 2.3 Laboratory tests

A complete blood count (hemoglobin and platelets) was obtained, and the levels of bilirubin, alanine aminotransferase (ALT), and aspartate aminotransferase (AST), and the international normalization ratio (INR) were determined. HCV RNA was detected by real-time qualitative PCR. HCV genotyping was not completed because of financial constraints.

### 2.4 Treatment protocol

All patients were started on sofosbuvir (200 mg for 6- to 11-year-olds and 400 mg for 12- to 18-year-olds) once daily and ribavirin (10–15 mg/kg/day) in two divided doses. The total period of therapy was 24 weeks, with genotype 3 requiring the most intensive therapy lasting at least 6 months [7,27].

### 2.5 Follow up

Patients were clinically followed up every four weeks in an outdoor setting, along with liver-function testing. Any history of adverse effects in response to a drug was recorded at each visit. A complete blood count, the levels of bilirubin, ALT, and AST, and the INR were determined on every follow-up visit. PCR was performed at 4, 12, and 24 weeks (at the end of treatment), and 12 weeks after the completion of treatment to detect a sustained virological response (SVR).

### 2.6 Operational definitions

Using real-time qualitative PCR, the efficacy of treatment was assessed in terms of the absence of HCV RNA. A negative PCR result four weeks into treatment was considered a rapid virological response. PCR positivity after 4 weeks of treatment and negativity after 12 weeks of treatment was considered an early virological response. When HCV was not detected in the blood 12 weeks or more after completing treatment, this was considered an SVR. Safety was outlined as the absence of significant side effects necessitating the termination of therapy.

### 2.7 Statistical analysis

The Statistical Package for Social Sciences version 24 was used to analyze the data. The qualitative variables included sex, different clinical presentations (jaundice, dark urine, abdominal pain, and hepatomegaly), chronic liver disease stigma, a virological response, and side effects. The quantitative variables included age, hemoglobin levels, total platelet count, total bilirubin, ALT and AST levels, and the INR. Statistical measures of the data, including the median, range, and percentages of different variables, were reported. To be statistically significant, *p*-values below 0.05 needed to be achieved.

## 3 Results

### 3.1 The basic characteristics of the reported cases

Twenty-six children were enrolled in the study aged between 6 and 18 years, with a mean age of 9.26 ± 2.82 years. A total of 7 (26.9%) children were aged 6–10 years, 10 (38.5%) were aged 11–15 years, and 9 (34.6%) were aged 16–18 years. Among the patients, 14 (53.8%) were male, and 12 (46.2%) were female (male-to-female ratio, 1.1:1).

### 3.2 Types of virological responses and the correlation with age

Twenty-five (96.1%) patients achieved an SVR with evidence of negative HCV RNA for at least 12 weeks of follow-up at the end of treatment. One patient (14.4%) was a nonresponder even after 24 weeks of treatment, and this patient belonged to the 6- to 10-year age group. Both the 6–10 and 11- to 15-year age groups had similar early and rapid virological responses. However, in the 16- to 18-year age group, most of the children achieved rapid virological responses (Table 1).

**Table 1:** Types of virological responses in different age groups (*n* = 26).

Virological response	6–10 years (*n* = 7)	11–15 years (*n* = 10)	16–18 years (*n* = 9)
Rapid	3 (42.8%)	5 (50%)	8 (88.8%)
Early	3 (42.8%)	5 (50%)	1 (11.2%)
No	1 (14.4%)	0 (0.0%)	0 (0.0%)
Sustained	6 (85.6%)	10 (100%)	9 (100%)

### 3.3 Clinical improvement after treatment

**Fig. 2:**
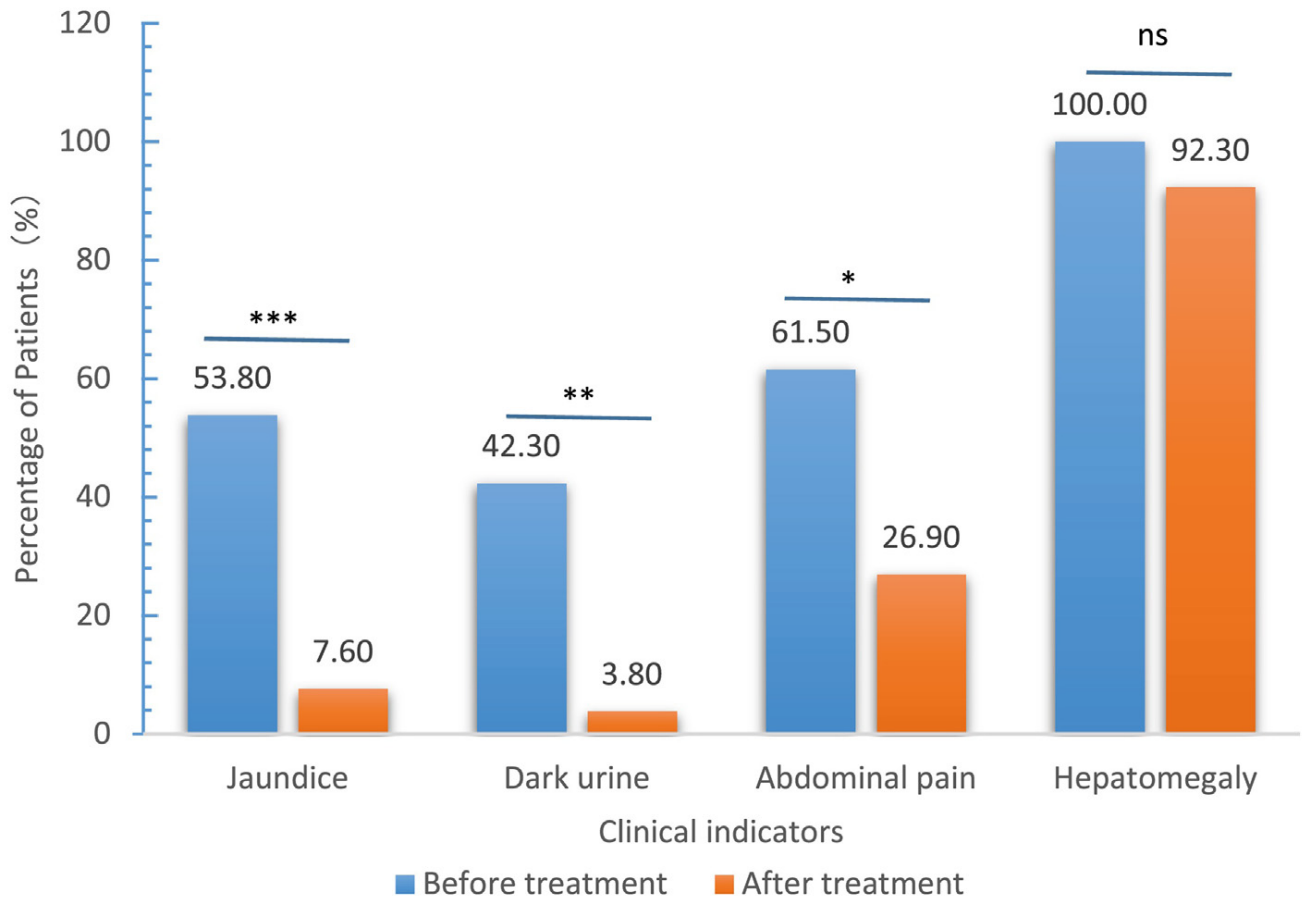
Clinical improvement following anti-viral therapy in patients with chronic hepatitis C virus infection (*n* = 26; ∗, *p* < 0.05; ∗∗, *p* < 0.01; ∗∗∗, *p* < 0.001; ns: no significance).

Patients showed significant improvement in jaundice, dark urine, and abdominal pain after treatment. Before treatment, jaundice, dark urine, and abdominal pain were present in 14 (53.8%), 11 (42.3%), and 16 (61.5%) cases, respectively, whereas after 6 months of treatment, they were present in only 2 (7.6%), 1 (3.8%), and 7 (26.9%) cases, respectively (Fig. 2).

### 3.4 Improvement in biochemical parameters and hematological status after treatment

Patients also showed a significant improvement in liver enzymes, as well as in serum bilirubin. Sofosbuvir and ribavirin therapy to treat chronic HCV infection caused a significant decrease in serum bilirubin from a median initial value of 3.2 (1.3–5.5) mg/dL to a value of 0.8 (0.5–3.2) mg/dL at final follow-up. Similarly, ALT and AST levels were also significantly decreased from median admission values of 212 (101–301) IU/L and 174 (63–212) IU/L to final follow-up values of 39 (24–222) IU/L and 30.5 (22–86) IU/L, respectively. The INR remained relatively constant from a median admission value of 1.2 (1.0–1.3) to a final follow-up value of 1.1 (0.9–1.2). Before and after therapy, hemoglobin (Hb) and platelet counts were also assessed. Hb dropped from a median starting value of 8.9 (7.8–11.1) mg/dL to 8.2 (7.1–10.6) mg/dL and platelets dropped from a median initial value of 127.5 (110–189) × 10^9^/L to 102 (85–156) × 10^9^/L at the final follow-up, albeit these changes were not statistically significant (Table 2).

**Table 2:** Median liver function and hematological test results following antiviral therapy in patients with chronic hepatitis C virus infection (*n* = 26).

Liver function and hematological indicators	Median (range) initial value (before treatment) *n* = 26	Median (range) final follow-up value (After treatment) *n* = 26	*p-* value
S. Bilirubin (mg/dL)	3.2 (1.3–5.5)	1.1 (0.5–3.2)	0.001
ALT (IU/L)	212 (101–301)	39 (24–222)	0.001
AST (IU/L)	174 (63–212)	30.5 (22–86)	0.001
INR	1.2 (1.0–1.3)	1.1 (0.9–1.2)	0.359
Hb (mg/dL)	8.9 (7.8–11.1)	8.2 (7.1–10.6)	0.382
Platelet ( × 10^9^/L)	127.5 (110–189)	102 (85–156)	0.184

ALT: alanine aminotransferase; AST: aspartate aminotransferase; INR: international normalization ratio; Hb: hemoglobin

**Fig. 3:**
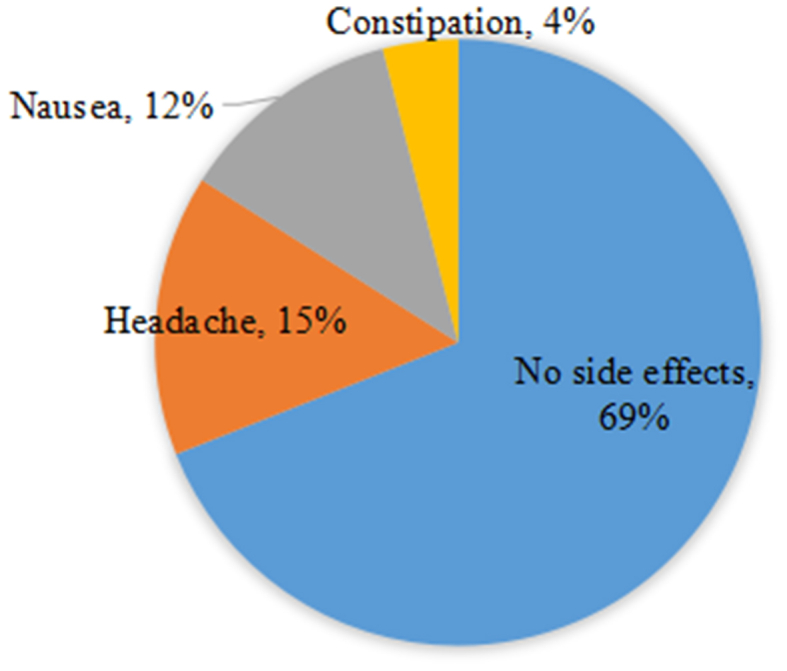
Side effects of treatment among the studied children (*n* = 26).

The treatment was well tolerated, with no evidence of side effects in 18 (69.2%) patients. No patients had serious side effects. A headache was reported in four (15.3%) patients, which gradually improved after 12–16 weeks of therapy. Three (11.5%) patients reported nausea, and one (3.8%) patient reported constipation that required a mild laxative (Fig. 3).

## 4 Discussion

The mean age of the children was 9.26 ± 2.82 years, which is similar to the mean age of children in other related studies [2,7]. The male-to-female ratio was 1.1:1 for Bangladeshi children, whereas it was 2:1 and 2.7:1 in similar studies in Pakistan and the USA, respectively [2]. Variability in the male-to-female ratio may reflect different sources of transmission.

This is the first study in Bangladesh to investigate the efficacy of antiviral agents in children and adolescents. Previous studies have reported that sofosbuvir and ribavirin have a high SVR (97%) [2,7,9]. In our study, the results were promising as the only patient who did not achieve SVR was a 6-year-old with a history of inconsistent medication intake because of financial constraints.

Our study found that the virological response developed more quickly among children in the 16- to 18-year age group than among younger children. The same outcome was seen in a study by Wirth et al. [9]. However, virological response developed more quickly as reported by Rosenthal et al. [7] in a study population of 3- to 12-year-olds. This variation in virological response may be caused by a better-developed immune response among older adolescents (16- to 18-year-olds), with higher antiviral activity inhibiting viral replication within 4 weeks of treatment.

In the present study, patients showed a significant (*p* < 0.05) improvement in jaundice, dark urine, and abdominal pain as well as in bilirubin, ALT, and AST levels. In another study, the same results were observed in Pakistani children [2]. An enhanced duration of treatment gradually reduces the viral load, allowing for improvements in liver function both clinically and biochemically. Hb and platelet counts also dropped from the median initial value to the final follow-up value, but this difference was not statistically significant. Studies by Urabe et al. [28] in Japan and Ghani et al. [29] in Egypt reported similar findings. In the present study, the regular blood transfusion of thalassemic children may be a key contributing factor.

The most frequently reported side effects were nausea and headaches, as well as vomiting [[7], [8], [9]]. Consistent with our findings, Wirth et al. found that the two most commonly reported adverse events were nausea (27%) and headache (23%) [9]. Rosenthal et al. [7] also observed similar adverse events, such as vomiting (32%) and headache (29%), and in a study in Pakistan, headache was observed in 8 (22.8%) patients. In the present study, treatment with sofosbuvir was generally safe and well tolerated. No side effects were observed in 18 (70%) patients. Four (15.3%) patients had headaches, three (11.5%) patients had nausea, and one (3.8%) patient had constipation that required a mild laxative.

The gold standard of HCV treatment has been interferon-based regimens. However, oral forms of DAA have added a new dimension to HCV treatment. As a direct consequence, treatments dependent on interferon are being rapidly phased out. However, available information on the effectiveness and potential adverse effects of DAA is primarily restricted to the adult population [30]. Patients with advanced conditions such as kidney failure and cirrhosis have greatly benefited from the use of these agents because of their low risk and high benefits [31]. The present study constitutes pioneering work on DAA in children and adolescents in Bangladesh as there was no previous data available regarding the pediatric population. Almost 96.1% of children achieved a virological response, and most of the patients achieved a rapid virological response. This research helps guide the therapeutic response to HCV infection in the future.

### 4.1 Limitations of the study

The study was a single-center study with a limited sample size because of the lack of health centers providing relevant treatment. In addition, viral genotyping was not completed because of financial constraints.

## 5 Conclusions

A combination of sofosbuvir and ribavirin is highly effective and well tolerated for chronic HCV infection in children with no major undesirable side effects. Sofosbuvir in combination with ribavirin provides an important option for treating chronic HCV infection in the pediatric population.

## 6 Recommendations

Genotype-specific DAA should be recommended for chronic HCV infection. However, if genotyping is not possible because of unavailability or financial constraints, then sofosbuvir with ribavirin may be recommended as an alternative. Further case-specific studies with larger sample sizes are recommended for a generalized conclusion.

## Funding

This research and the publication were completely funded by the authors.

## Author contributions

SM conceived the study, participated in its design and coordination, and prepared the first draft of this paper. JG and MRM worked on the design of the study and performed the statistical analysis, as well as the interpretation of the results. BH, MB, RR, FT, ARH, TF, and JA took part in the design, coordination, and preparation of the manuscript. SSA was involved in the overall supervision of the study, as well as in critically reviewing the manuscript for important intellectual content. All of the authors read and approved the final version of the paper.

## Acknowledgments

The authors thank all of the participants, their parents, and the institute for their support and contributions.

## Declaration of competing interest

The authors declare that they have no known competing financial interests or personal relationships that could have appeared to influence the work reported in this paper.

## Data available statement

The patient data that support the findings of this study are available on request from the corresponding author. The data are not publicly available because of privacy or ethical restrictions.

## Ethics statement

Ethical clearance was obtained from the ethical review committee of Bangladesh Shishu Hospital & Institute (No. Admin/1041/DSH/2021).

## Informed consent

Written informed consent was obtained from the parents of individual participants at the time of admission.
